# Computer vision for plant pathology: A review with examples from cocoa agriculture

**DOI:** 10.1002/aps3.11559

**Published:** 2023-12-19

**Authors:** Jamie R. Sykes, Katherine J. Denby, Daniel W. Franks

**Affiliations:** ^1^ Department of Computer Science University of York Deramore Lane, York YO10 5GH Yorkshire United Kingdom; ^2^ Centre for Novel Agricultural Products, Department of Biology University of York Wentworth Way, York YO10 5DD Yorkshire United Kingdom; ^3^ Department of Biology University of York Wentworth Way, York YO10 5DD Yorkshire United Kingdom

**Keywords:** agronomy, disease detection, machine learning, plant pathology

## Abstract

Plant pathogens can decimate crops and render the local cultivation of a species unprofitable. In extreme cases this has caused famine and economic collapse. Timing is vital in treating crop diseases, and the use of computer vision for precise disease detection and timing of pesticide application is gaining popularity. Computer vision can reduce labour costs, prevent misdiagnosis of disease, and prevent misapplication of pesticides. Pesticide misapplication is both financially costly and can exacerbate pesticide resistance and pollution. Here, we review the application and development of computer vision and machine learning methods for the detection of plant disease. This review goes beyond the scope of previous works to discuss important technical concepts and considerations when applying computer vision to plant pathology. We present new case studies on adapting standard computer vision methods and review techniques for acquiring training data, the use of diagnostic tools from biology, and the inspection of informative features. In addition to an in‐depth discussion of convolutional neural networks (CNNs) and transformers, we also highlight the strengths of methods such as support vector machines and evolved neural networks. We discuss the benefits of carefully curating training data and consider situations where less computationally expensive techniques are advantageous. This includes a comparison of popular model architectures and a guide to their implementation.

Computer vision (CV), typically powered by machine learning (ML), is now used for a variety of tasks in agriculture, botany, and ecology. These tasks include plant health assessments (Patrício and Rieder, 2018), identification of weeds (Wu et al., 2021), identification of drought‐prone areas of land (Ramos‐Giraldo et al., 2020), yield prediction (Sarkate et al., 2013), and detection of defects or bruising in fruits and vegetables (Tripathi and Maktedar, 2020). We are seeing substantial improvement in the efficiency of CV techniques (He et al., 2016; Howard et al., 2017; Zhang et al., 2018) and, at least for now, computational resources continue to become more affordable (Mack, 2011). As a result, CV is becoming available to whole industries, not just areas of highest commercial value; for example, ML has been used with increasing regularity for tasks specific to cocoa (*Theobroma cacao* L.), such as the exploration and optimisation of aroma profiles (Fuentes et al., 2019), monitoring of cocoa bean fermentation (Parra et al., 2018; Oliveira et al., 2021), and bean quality classification (Mite‐Baidal et al., 2019). While large research and development budgets for areas such as wheat (*Triticum aestivum* L.) production have allowed for the use of unpiloted aerial vehicle photography to identify disease outbreaks (Su et al., 2018; Chiu et al., 2020) and the use of multispectral satellite photography to monitor outbreaks of yellow rust (*Puccinia striiformis*) from space (Nagarajan et al., 1984), the application of ML to sectors with fewer financial resources has had to take a different form. Onboard graphics processing units (GPUs) can run large neural networks locally, analysing image data from farm machinery in real time, while fast internet connections can be used to run the same large models remotely (Grosch, 2018). By contrast, implementation of ML in poorer sectors must rely on older hardware, edge devices, and older‐model smartphones. This means that an emphasis must be placed on the ultra‐low‐cost implementation and high computational efficiency of algorithms. This provides us with an opportunity and motivation to steer the ML field away from brute force computing and toward more nuanced and efficient approaches.

The cultivation of cocoa represents a prime example of a sector that could benefit greatly from non‐intrusive and highly optimized CV disease detection and will be used as an example throughout this review. The International Cocoa Organization estimates that up to 38% of the global cocoa crop is lost to disease annually, with over 1.4 million tonnes of cocoa lost to just three diseases in 2016 (Maddison et al., 1995; Marelli et al., 2019). Additionally, international disease spread has been devastating to this industry in the past and could be again in the future (Phillips‐Mora and Wilkinson, 2007; Meinhardt et al., 2008). Following the loss of a cocoa crop to witches’ broom disease, a plot of land will typically be cleared of forest, and the previous robust agroforestry system will be replaced with a monoculture (Rice and Greenberg, 2000; Meinhardt et al., 2008). This disease is therefore not only capable of devastating the livelihoods of whole communities of cocoa farmers, eliminating 50–90% of their crop (Meinhardt et al., 2008), but it is also destructive to local biodiversity and has significant negative impact on the carbon capture potential of the land (Kuok Ho and Yap, 2020). Such loss of Amazonian Forest is a driver of climate change, causing positive feedback and exacerbating this global crisis (Malhi et al., 2008).

A review from 1986 on the use of systemic fungicides to tackle oomycetes, such as *Phytophthora* spp., highlights concern about damage to the environment and human health by pesticides such as methyl bromide, which are still in use today (Cohen and Coffey, 1986). These concerns, and those of the pesticide resistance (Department of Health, Victoria, 2018), are still present 37 years later. The use of CV and ML for targeted application and calibration of pesticide dose are beginning to have massive beneficial effects in this area across the agriculture industry.

It is estimated that from 2016 to 2026, the number of smartphone users will have doubled from approximately 3.7 billion people to 7.5 billion (Statista, 2022). Therefore, the necessary hardware to run CV models is largely in place, and we need now only develop and deploy the CV models to have great potential for impact with little monetary input. Here we discuss how best to achieve this.

This review is composed of three main sections. Section 1, “Methods in computer vision,” critically reviews a wide variety of relevant techniques in ML and CV model development and testing, and section 2, “Data acquisition and model testing,” discusses techniques for data gathering, data labelling, and model testing. While section 1 focuses on ML theory and comparison of model architectures, section 2 focuses on more practical issues. The final section, “A roadmap to commercial implementation,” includes multiple points that are important to consider prior to choosing an architecture and beginning development.

Several review articles have been published on the topic of CV and deep learning that are applicable to plant pathology (Voulodimos et al., 2018; Weinstein, 2018; Chouhan et al., 2020; Xu et al., 2021). High‐quality works such as Weinstein (2018), which reviews the use of CV in animal ecology, are directly applicable to plant pathology owing to the flexibility of the techniques discussed here. What is missing from these works is a critical review and discussion of the latest and/or less conventional techniques in CV and a discussion of data acquisition and validation. Each of these reviews were published prior to or near the release of Detection Transformer (DETR; Carion et al., 2020), Vision Transformer (VIT; Dosovitskiy et al., 2021), and ConvNeXT (Liu et al., 2022), so naturally these recent landmark methods are not discussed. However, despite all being published after the release of Faster Region‐Based Convolutional Neural Network (Faster R‐CNN; Ren et al., 2016), ResNet (He et al., 2016), and You Only Look Once (YOLO; Redmon et al., 2016), only Xu et al. (2021) mention any of these popular and high‐performing architectures. Those being YOLO and region‐based fully convolutional networks, an early predecessor to Faster R‐CNN.

A recent survey (Guo et al., 2022) goes into great detail on the various facets of different attention mechanisms, which are integral to transformer architectures. While this work presents the bleeding edge of CV technology, it does not present the holistic, applied, and data‐centric perspective provided here. Another paper aimed to develop CV models for the classification of cocoa beans, comparing the use of ResNet18, ResNet50, and support vector machines (SVMs; Lopes et al., 2022), while another recent review gives a high‐level discussion of a number of CV studies in agriculture, covering the topics of hyperspectral imaging, the use of unpiloted aerial vehicles, and architectures as recent as ResNeXt (Xie et al., 2017; Tian et al., 2020). However, while the latter of these two papers presents a broad view of CV for plant pathology, providing strong links to many plant taxa, no mention is made by either Lopes et al. (2022) or Tian et al. (2020) of architectures or techniques released after 2017. As such, the fusion of industry standard and bleeding edge methods in data acquisition, verification, and analysis presented here make the present review unique among those listed above.

This review provides the reader with an in‐depth understanding of CV for plant pathology and supports the previous works. In doing so, we focus on how best to adapt current methods to provide practical solutions for farmers, agronomists, and botanists without access to high‐performance computational resources. While cocoa agriculture is used as a consistent example throughout, all methods discussed here are applicable across plant pathology and agriculture, as well as related fields such as plant and animal ecology and forestry.

## METHODS IN COMPUTER VISION

### Background

Ever since AlexNet was presented at the Conference on Neural Information Processing Systems in 2012, the field of CV has been dominated by CNNs (Krizhevsky et al., 2017). While subsequent updates to CNN architectures have provided dramatic improvements over AlexNet (Liu et al., 2022), it is important to recognise that CNNs are not the only tools at our disposal. Previous work on cocoa disease has assessed the performance of SVMs, random forest regression, and artificial neural networks to identify common diseases in cocoa from standard colour images, hereafter referred to as RGB (red, green, blue) images (Rodriguez et al., 2021). Here it was shown that artificial neural networks are capable of identifying late‐stage disease in RGB images of cocoa, but that training data set size is a limiting factor. Another study applied an SVM to perform pixel‐wise identification of black pod rot in cocoa (Tan et al., 2018). The resulting algorithm showed an impressive ability to detect human‐visible disease symptoms and, given the high computational efficiency of SVMs, it was able to run on low‐powered hardware. Additionally, this model was trained on only 50 images, which is an extremely small training set in CV. However, no mention was made of the ability of these models to detect early disease development or non‐human‐visible symptoms, which will be a central focus of this review.

### Vision transformers

In the early 2010s, transformers become the default for natural language processing (Liu et al., 2022), and they are now rapidly gaining popularity in vision‐based tasks. Pure transformer‐based multilayer perceptrons, such as ViT (Dosovitskiy et al., 2021), do away with the convolutional layers of a CNN. Instead, they subdivide and tokenise an image, giving each token a positional embedding, and then pass all of these data to the multi‐head attention mechanism of the network. The main drawbacks of such transformer‐based models are that they require training data sets on the order of millions of images, and they lack the inductive biases of CNNs, such as translational equivariance (Dosovitskiy et al., 2021). In addition, the global structure of objects in an image must be learned from scratch, whereas this is maintained throughout a CNN. However, when pretrained on a large data set and then fine‐tuned on a more modest data set of tens of thousands of images, vision transformers can outcompete CNNs (Dosovitskiy et al., 2021).

Although the requirement for vast training data sets may preclude the use of transformers for many plant pathology projects, there is a middle ground between the popular ResNet architectures and transformer models. Taking inspiration from transformer designs, the highly competitive ResNet architectures were updated to produce a pure CNN that competes well with transformers in many tasks and is reported to outperform the original ResNets by about 3% accuracy on ImageNet (Deng et al., 2009). This family of four models is named ConvNeXt and includes models of varying complexity from ConvNeXt Tiny to ConvNeXt Large. Additionally, ConvNeXt uses layer normalisation in place of batch normalisation. This modification could have important benefits for plant pathology projects, as discussed in the “Image, batch, and layer normalisation” section; however, as the ConvNeXt architectures are relatively large (ConvNeXt Tiny: 29 million parameters, ResNet18: 12 million parameters, ResNet50: 26 million parameters), these models too require large and/or complex training data sets to avoid overfitting and more powerful hardware to run at inference than the smaller ResNets.

### Object detection and semantic segmentation

Bounding box object detection and semantic segmentation are processes by which objects of interest in an image are both classified and located in the image. In these tasks, either a box (bounding box object detection) or a polygon or “mask” (semantic segmentation) is drawn around the object of interest. For an example of semantic segmentation, see Case Study 1 (Box 1).

Box 1 **Case Study 1: Semantic segmentation for cocoa disease detection.**

In this case study, we applied Mask R‐CNN to segment images of diseased cocoa trees. The training data set consisted of 186 images of black pod rot, 121 images of frosty pod rot, and 63 images of witches’ broom disease. The model was trained, starting with the “mask rcnn R 50 FPN 3x” weights, for 1000 epochs.

**Figure 1:**
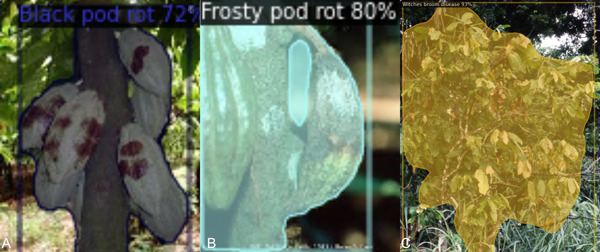
Application of semantic segmentation with Mask R‐CNN to highlight whole diseased cocoa trees. Example images of trees infected with (A) black pod rot, (B) frosty pod rot, and (C) witches’ broom disease. The percentage scores for each show the degree of confidence in the model's diagnosis: black pod root = 72%, frosty pod root = 80%, witches’ broom disease = 93%.

The preliminary results from this case study were somewhat encouraging. However, although the selected positive results in Figure 1 show that this model has the potential to perform well, these results are not representative of the full testing set. The average precision per class was 4.29, 13.45, and 30 for black pod root, frosty pod root, and witches’ broom disease, respectively, i.e., the model performed acceptably on witches’ broom disease, despite the low number of training images, but poorly on most cases of black pod root and frosty pod root.

Notwithstanding the potential theoretical benefits discussed above, manual annotation of a full training data set with masks is extremely laborious. So, without the promise of improved results relative to a simple CNN, this additional effort may not be worthwhile. Despite this, the favourable preliminary results in this study and one other (Zhao et al., 2018) mean that, with the incorporation of automated annotation tools and/or semi‐supervised learning, semantic segmentation shows promise as an avenue of research for CV in plant pathology.

Semantic segmentation and object detection could help in the accurate manual labelling of disease states in images. In simple image classification with a CNN, a model must learn what features, across the whole image, can be used as true markers of disease. However, annotation of training images with bounding boxes or segmentation masks may be used to focus the attention of the model, thus making training more efficient and reducing overfitting. This beneficial effect might be more pronounced with semantic segmentation than bounding boxes because the edges of a bounding box may extend beyond the edges of the leaf, pod, or tree in question and thus mislabel parts of neighbouring healthy plants. However, when comparing the ability of Faster R‐CNN and Mask R‐CNN to detect human‐visible signs of insect damage in sweet peppers (*Capsicum annuum* L.), Faster R‐CNN was shown to have superior accuracy and mean average precision (mAP) (Lin et al., 2020). Here, mAP is defined as the mean precision over all classes of the mean per‐class precision, with a given intersection over union. These disparities in performance were contingent on which backbone model architecture (Inception v2, ResNet50, or ResNet101) (Szegedy et al., 2016) was used. When the more complex ResNet101 was used, Faster R‐CNN and Mask R‐CNN performed more similarly, although in this task Faster R‐CNN performed best with the simpler architectures (Lin et al., 2020). However, it should be noted that average precision is not directly comparable between bounding box detection and semantic segmentation models. This is for two reasons: (1) it is easier to achieve a given intersection over union with a bounding box as this task is less precise than segmentation, and (2) Mask R‐CNN simply adds the ability to predict a mask in a box predicted by Faster R‐CNN, so segmentation is additive in this case. As such, the results of Lin et al. (2020) should be considered accordingly.

Object detection and semantic segmentation are typically performed using Faster R‐CNN (Ren et al., 2016), Mask R‐CNN (He et al., 2017), or YOLO (Redmon et al., 2016). However, these architectures have also been combined with other methods, such as SVMs, to confirm or deny the presence of an object in a proposed region (Voulodimos et al., 2018). For example, SVMs have been used in conjunction with Mask R‐CNN in automated ML pipelines to identify defects in machined parts (Huang et al., 2019). Additionally, when facing a classification problem with high intraclass variance, low interclass variance, and insufficient training examples, the application of SVMs to features learned by a CNN from ImageNet can improve results relative to a CNN alone (Cao and Nevatia, 2016). This may prove useful in projects with few training images, or when classifying images of plant disease with similar characteristics, such as black pod rot in cocoa caused by *Phytophthora megakarya* or *P. palmivora*. Furthermore, while *P. megakarya* and *P. palmivora* can be distinguished by eye, *Lasiodiplodia* species, of which three are known to infect cocoa, can present with identical morphological characteristics. This means that traditional classification techniques are insufficient and molecular identification techniques must be used in their place (Huda‐Shakirah et al., 2022). The development of CV technologies that can make such difficult distinctions would have important implications for all areas of agriculture and botany for two reasons. First, while *P. megakarya* and *P. palmivora* are managed in the same way, different species of *Lasiodiplodia* are not (Khanzada et al., 2005). Thus, the failure of a model to distinguish between species of *Phytophthora* is not critical for effective disease management, but failure to distinguish between species of *Lasiodiplodia* is. Second, cosmopolitan pathogens such as *Phytophthora* spp. and *Lasiodiplodia* spp. have extremely wide host ranges, infecting many commercially important crops. *Lasiodiplodia theoromae* alone attacks over 189 plant species across 60 families (Salvatore et al., 2020), while the growing list of described *Phytophthora* (aka “plant destroyer”) species is currently 116 entries long (Kroon et al., 2012).

Transformer‐based object detection models such as DETR (Carion et al., 2020) are also now available and contend well with Faster R‐CNN when trained on the huge Common Objects in Context (COCO; Lin et al., 2014) benchmark data set. The key benefit of DETR is that it predicts bounding box coordinates directly, negating the need for the region proposal network of Faster R‐CNN. Faster R‐CNN's region proposal network has issues trying to identify overlapping objects because of the non‐max suppression algorithm, which was removed from YOLO in version 3 (Horzyk and Ergün, 2020). However, DETR has problems detecting small objects, and has a very long convergence time. These defects are said to be resolved in Deformable DETR (Zhu et al., 2021), although we encountered significant difficulty in retraining Deformable DETR due to prevailing bugs in the code and so were unable to confirm these benefits.

In segmenting instances of nuclei in microscopy images, Mask R‐CNN was compared with the U‐Net architecture (Ronneberger et al., 2015), which was designed for medical image segmentation. Here, the two techniques were shown to give similar mAP, F1, and recall scores (Vuola et al., 2019), although Mask R‐CNN scored 0.812 for precision, while the U‐Net scored only 0.68. A subsequent ensemble approach was then described, which shares the outputs of the two independently trained architectures to exploit the U‐Net's purportedly superior F1 scores (+0.057), in tandem with Mask R‐CNN's high mAP, precision, and recall. The ensemble model produced comparable, if slightly higher, mAP (+0.016), F1 (+0.056), and recall (+0.037) scores compared with Mask R‐CNN, but the precision was 0.087 lower. Although the U‐Net was reported to produce the best F1 score and the ensemble model produced the best mAP and recall, these improvements were slight. Additionally, F1 is calculated directly from precision and recall, so it seems counterintuitive that the U‐Net approach could have the highest F1, yet the lowest precision and recall. The most noteworthy result here is the consistently superior precision of Mask R‐CNN in this comparison and in another against YOLO (Bharati and Pramanik, 2020; Horzyk and Ergün, 2020). Additionally, in a study comparing the use of U‐Net and Mask R‐CNN to segment images of pomegranate (*Punica granatum* L.) trees, Mask R‐CNN outperformed the U‐Net in both precision and recall by wide margins (Zhao et al., 2018).

An alternative approach applied an SVM to perform pixel‐wise classification to detect black pod rot in cocoa, with a human expert labelling the diseased pixels in training images (Tan et al., 2018). Like semantic segmentation, this technique achieves the effect of providing the model with additional information on the location of disease in an image, relative to a simple CNN. However, it imposes arbitrary physical boundaries around disease symptoms such as lesions and cankers, and the algorithm is unable to define for itself any symptoms that are not or cannot be identified with human vision. By using semantic segmentation with a CNN backbone, like in Mask R‐CNN or DETR, to segment whole trees, these effects could be avoided, i.e., the model would be able to detect non‐human‐visible symptoms via feature learning and model the effects of hyphae propagating through the plant or systemic changes to a plant's phenotype away from the site of infection.

### Variational autoencoders for outlier detection

In addition to discriminative modelling, ML provides several powerful tools for generative modelling. Modelling with generative deep neural networks (DNNs) can aid in gaining an intuitive understanding of the physical laws that led to the creation of the data to be modelled. An example of this is the use of artistic style transfer with generative adversarial networks (Li and Wand, 2016), where specific semantic features in an image can be isolated and utilised. Another popular deep generative model architecture is the variational autoencoder (VAE), which we will focus on here for the task of image data set filtering.

When working with autonomously collected data, for example from camera traps or web‐scraping bots, the acquisition of vast quantities of data is often the easy part of creating a good training data set. Camera traps tend to produce a considerable amount of uninformative data and the data from naive web‐scraping bots can be badly contaminated with misclassified and irrelevant images; for example, a search for the keyword “Acer” will return many more images of laptops than it will Japanese maple trees, and a search for “black pod rot” will include many images of frosty pod rot, cherelle wilt, and insect damage. Therefore, some level of human supervision is vital in curating training data, and the importance of consulting farmers and researchers in data collection and labelling cannot be overstated. However, manual labelling of a full data set can be extremely costly, and a potential method to offset some of this cost is said to be the use of VAEs for outlier detection.

A VAE is composed of two neural networks that are trained in parallel. The encoder network projects the image data to a smaller latent vector space, thus compressing it, and the decoder network predicts the original image from this compressed data as best it can.

Generative models tend to generalise to the real world much better than discriminative models, which aim to uncover correlative relationships between data and class labels (Kingma and Welling, 2019). However, deep generative models are typically considered excessive for classification problems, as they often have higher bias (Banerjee, 2007) and are computationally expensive.

Previous works have successfully used VAEs for text classification (Xu et al., 2017; Xu and Tan, 2020), data clustering (Dilokthanakul et al., 2017; Lim et al., 2020), anomaly detection (An and Cho, 2015), recommender systems (Li and She, 2017), and dimensionality reduction (Lin et al., 2020). There are also a limited number of published papers on the use of VAEs for anomaly detection with colour images (Fan et al., 2020).

Here, we consider two methods for which a VAE might be used to detect outlying data in collections of large colour images. To do so, we will use the example of detecting non‐plant images in a web‐scraped collection of plant images for use in building a disease classifier.

**Method 1. Distribution of reconstruction loss**

Having trained a VAE on only plant images, use this model to compress and decompress all images in the contaminated data set and record the reconstruction loss for each image. Plot the distribution of the loss values and record the most extreme high values as outliers. The assumption here is that the model should “fail” to reconstruct non‐plant images well, as it should be naive to any images that do not show plants.

**Method 2. Dimension reduction and clustering**

Using the encoder network of a VAE that has been trained on the ImageNet data set, compress the images in the contaminated data set and record the values of the latent space for each image. Reduce the dimensions of the latent space further with principal component analysis, t‐distributed stochastic neighbour embedding (t‐SNE), and/or uniform manifold approximation and projection (UMAP). Plot these reduced data. Outliers/contaminant images may then separate from the clean data.

Nouveau VAE (NVAE) is the product of an effort to carefully craft the encoding network architecture of a VAE, which appears to produce excellent results (Vahdat and Kautz, 2021). After training for just one epoch, this architecture is able to project large colour images onto a latent space and reconstruct them almost perfectly. However, if the aim of using NVAE is to compress image data, this architecture is not appropriate. This is because, using the recommended settings for the CelebA 64 data set (Liu et al., 2015), the latent space produced for an image with dimensions (3,224,224) is (100,224,224), i.e., more than 33 times larger than the original image. Following the authors’ provided instructions to constrain the latent space to be as small as possible without excessively modifying the code, the latent space for this same size of image remains the same (100,224,224). This observation is corroborated in another study where the authors explain how NVAE first expands the data dimensions to a large number of latent spaces before pruning those spaces based on Kullback–Leibler (KL) divergence (Asperti et al., 2021). However, these authors go on to note that, in their use case, NVAE transformed images of size (3,32,32) to a latent space of size (16,16,128) without any subsequent downscaling. It is not surprising then that this architecture is able to reconstruct an image so well after just one training epoch, with no pre‐trained weights, as the dimensionality of the data is expanded rather than compressed. Likewise, NVAE is not appropriate for identifying outliers by the distribution of reconstruction errors as it can reconstruct any image almost perfectly. For example, when we trained NVAE on a data set of 54,124 plant images, it was able to reconstruct any image in the ImageNet data set with similar binary cross‐entropy loss to that of plant images. As an alternative to NVAE, we attempted to use a custom convolutional VAE with a ResNet152 (He et al., 2016) backbone to apply the two methods of outlier detection described above. However, we were unable to get this architecture to function well enough to sufficiently compress the data and reconstruct images with high fidelity.

The paucity of papers published on the subject of outlier detection in colour images with VAEs seems to be due to the inherent difficulty of this task. The high dimensions of such data and the large storage and GPU memory requirements that training these models on such data necessitates (Sun et al., 2018) has largely been resolved, although for many projects GPU memory availability will still preclude this technique. Thus far, the inability of the VAE architecture to learn a compression algorithm for large colour images suggests a hard physical limitation that might not be overcome. Moreover, while Maalø et al. (2019) contest this argument, Nalisnick et al. (2019) argue comprehensively that generative models are not suitable for outlier detection by the reconstruction loss method described above, as these models tend to learn low‐level statistics about data rather than high‐level semantics. As such, they are often unable to differentiate between images that, to the human eye, are obviously different. We describe a successful alternative method of outlier detection in Box 2.

Box 2 **Case Study 2: Semi‐supervised learning for outlier detection.**

As an alternative to using a variational autoencoder for outlier detection, we trained a semi‐supervised binary Outlier‐NoneOutlier (in this case, “plant” or “non‐plant”) classifier, which achieved near‐perfect results. We used the ResNet18 architecture and initially trained it on a manually curated data set of 57,228 plant images and an equal‐sized random subset of the ImageNet data set, which constituted the non‐plant images. We then continued training using the below algorithm and the contaminated data set of 96,692 images.

**Table:** 

**while** *nRelabledImages* > 0 **do**
**train model**
**for image in ContaminatedImages do**
**classify image**
**if** *ClassificationConfidence* ≥ 99% **then**
**label image**
add image to training set
**end if**
**end for**
**end while**

During this process, 1376 non‐plant images and 44,212 plant images from the contaminated data set were correctly labelled by the model. After the first round of semi‐supervised training was completed, images that this model classified with >99% confidence were manually reviewed. Incorrectly labelled images were manually re‐labelled and a second round of semi‐supervised training was begun. After the first round of semi‐supervised training, classification of images as “plant” with >99% confidence was >99% accurate but classification of images as “non‐plant” with >99% confidence was only about 50% accurate. After the second round of semi‐supervised training, the model performed with >99% accuracy and F1 score for both classes, thus showing a clear superiority in this technique's ability to identify contaminant images over the VAE approaches. This is in addition to its ease of implementation, reduced training time, and low computational requirements. After training, the model was used to classify all 96,692 images in the contaminated data set.

### Evolutionary algorithms

The field of CV is currently dominated by handcrafted DNNs with fixed topologies. However, the seldom‐used techniques of evolved neural networks have real potential in the field of plant pathology. Computational efficiency at inference and improved ability to generalise are of paramount importance to models developed for plant pathology in the field. This is because such models must be able to cope with complex and highly variable symptoms and backgrounds, and often must run on low‐powered hardware. Growing neural networks take far longer to train/grow than those with fixed topologies, but this is of minor concern given the efficient parallelisation and the vast computational resources now available for training. The hardware available to farmers in low‐income sectors, such as cocoa, cassava (*Manihot esculenta* Crantz), or coffee (*Coffea* L. spp.) cultivation, is restricting. This restriction means that producing a model that is optimised for runtime speed at inference is a vital factor, and growing neural networks with evolutionary algorithms may be an ideal way to achieve this.

Evolving neural networks have been shown to be highly effective in producing neural networks with a high degree of modularity (Amer and Maul, 2019). This increased modularity is said to be the result of applying a cost to the number of connections, which both reduces computational cost and promotes evolvability as the sharing of modular units between parents is made simpler. It is also said that such modularity helps these models to generalise better as each modular unit is capable of independent generalisation (Schmidt and Bandar, 1998). With evolutionary algorithms, one can also promote diverse populations of networks with techniques such as niching (Shir, 2012), and use of non‐elitism strategies can allow for the simultaneous exploration of fitness valleys and local optima without getting stuck there (Dang et al., 2021). While elitism follows the biologically implausible assumption that the fittest individual/network will always survive to reproduce, non‐elitism allows weaker individuals to explore fitness valleys, which may lead them to undiscovered maxima.

While a direct comparison of evolved neural networks with popular CNN architectures could not be found, Table 1 shows an indirect comparison between a recent method for evolving neural networks (EVOCNN) and two popular CNNs, ResNet18 and VGG16; EVOCNN appears to perform very well in this comparison. However, the error rate for these models was calculated when trained on the Fashion‐MNIST data set, while the top 1 and top 5 accuracy was produced using ImageNet. Fashion‐MNIST, which is composed of 28 × 28‐pixel greyscale images of clothing (Xiao et al., 2017), is not a challenging proposition for modern CNNs and is not reflective of real‐world plant pathology problems. Additionally, it should be noted that, in the EVOCNN paper (Sun et al., 2020), the number of parameters of VGG16 is misreported as 26 million, rather than the 138 million listed in the Torchvision documentation (PyTorch, 2023). This suggests that VGG16 would have massively overfit to the Fashion‐MNIST data, making this an inappropriate comparison. However, EVOCNN does offer a very low error rate on this simpler problem and has a very low number of parameters when compared with other modern architectures (Tables 1 and 2). Overall, it seems that evolved neural networks are not yet ready to tackle the more difficult problems in plant pathology, and so more work is required in this area.

**Table 1:** Test results of three architectures trained on two data sets, providing an indirect comparison. ResNet18 was trained only on ImageNet with the top 1 and top 5 classification accuracies shown. EVOCNN was trained only on Fashion‐MNIST with the percent error shown. VGG16 was trained on both data sets. Results were taken from Sun et al. (2020) and the PyTorch documentation (PyTorch, 2023). Boldfaced text signifies the best result per metric.

Architecture	Top 1 accuracy (%)	Top 5 accuracy (%)	Error (%)	No. of parameters
ResNet18	69.758	89.078	—	11.7 M
VGG16	**71.59**	**90.38**	13.78	138 M^a^
EVOCNN	—	—	**7.28**	**6.52 M**

^a^ Number of parameters for VGG16 was misreported by Sun et al. (2020) as 26 million.

### Architecture comparison and recommendations

The field of CV has produced a numerous and diverse set of architectures, each with unique strengths and weaknesses. Here, we will compare these architectures, focusing on their application in image classification, object detection, and semantic segmentation. Table 2 gives a detailed breakdown of the pros and cons of each of these architectures, as well the number of trainable parameters, which acts as a proxy for model complexity, and the number of giga floating point operations (GFLOPS), which gives a sense of computation cost of running inference with these architectures.

**Table 2:** Pros and cons of popular model architectures for image classification, object detection, and semantic segmentation. Ranges of values represent the smallest and largest off‐the‐shelf versions available.

	Architecture	No. of parameters	GFLOPS	Pros and cons
**Image classification**	ResNet^a^ (2015)	12–60 M	1.8–11.5	**Pros** ResNet18 is the smallest and most computationally efficient model here ResNet18 is ideal for modestly sized data sets ResNet152 performs comparably with transformers such as VIT Widely used and tested
				**Cons** Uses batch normalisation, which can introduce instability and inconsistent results
	EfficientNet‐V2^a^ (2019)	22–119 M	8.4–56.1	**Pros** Allows the depth, width, and resolution of the model to be scaled with a single coefficient
				**Cons** Scaling requires editing the source code Evaluation using Grad‐CAM (Selvaraju et al., 2017) showed much overfitting, despite high test scores
	ConvNeXT^a^ (2022)	29–198 M	4.5–34.3	**Pros** Reported to outperform any architecture here and requires much less data than VIT Scaled easily by editing the convolutional block settings Incorporates several modern features such as GELU, stochastic depth, and layer normalisation
				**Cons** The smallest off‐the‐shelf configurations are too large for many projects and may overfit Potential compatibility issues with conversion to ONNX format
	ViT^a^ (2021)	87–634 M	17.6–1016	**Pros** If trained on millions of images, VIT may slightly outperform ResNet152
				**Cons** Requires huge data sets to outperform CNNs Computationally expensive to train and run at inference
**Object detection and semantic segmentation**	Faster R‐CNN^a^ (2015)	44 M	280.4	**Pros** Generally gives higher mean average precision than YOLO Performs better than YOLO on small objects
	Mask R‐CNN^a^ (2017)	46 M	333.6	**Cons** More computationally expensive than YOLO Does poorly when objects overlap
	YOLO^b^ (2016)	7 M	1.01	**Pros** Extremely fast inference time Fast to train Very easy to implement
				**Cons** Performs poorly on small objects Gives the least accurate results of the three architectures listed here
	DETR^b^ (2021)	40 M	11.2	**Pros** Negates the need for region proposal and non‐max suppression Performs better than Faster R‐CNN and YOLO for overlapping objects As opposed to classification, transformers such as DETR show promise in object detection Faster at inference than Faster R‐CNN
				**Cons** Very computationally expensive to train Slow to converge in training Requires huge amount of training data Can be challenging to implement Requires a large batch size to achieve stable training

*Note*: CNN = convolutional neural network; DETR = Detection Transformer; Faster R‐CNN = Faster Region‐Based Convolutional Neural Network; GELU = Gaussian error linear unit; GFLOPS = giga floating point operations; Mask R‐CNN = Mask Region‐Based Convolutional Neural Network; ResNet = Residual Neural Network; ViT = Visual Transformer; YOLO = You Only Look Once.

^a^ Values for the number of trainable parameters (displayed in millions [M]) and GFLOPS were obtained from the PyTorch documentation (PyTorch, 2023).

^b^ Values for the number of trainable parameters (M) and GFLOPS for YOLO and DETR were calculated using an image 224 × 224 pixels for this comparison.

#### Image classification architectures

ResNet introduced the concept of skip connections, enabling the training of much deeper models. Despite its age, ResNet remains a strong competitor, and ResNet18 is probably still the best choice for most small projects with fewer training examples. EfficientNetV2 (Tan and Le, 2021) is more computationally demanding than equivalent ResNet and ConvNeXT variants, and while it tends to yield high accuracy on large data sets (Dosovitskiy et al., 2021; Liu et al., 2022), we found that it is prone to overfitting, making it a less favourable choice. The key innovation of EfficientNet was to allow the depth, width, and resolution of the model to be scaled by adjusting a single coefficient (Tan and Le, 2020). However, in practice this requires editing the source code, thus rendering such adjustments less than convenient. ConvNeXT is an updated version of ResNet, incorporating several modern features. Unlike EfficientNet, ConvNeXT is easy to scale, making it a promising choice for medium‐ to large‐scale applications, for which it has been shown to give superior performance to ResNet and VIT (Liu et al., 2022). As the first transformer to perform favourably against CNNs for image classification, VIT represents a significant milestone. However, image classification may not be the optimal use case for transformer architectures, and at present ConvNeXT outperforms VIT while requiring less data for training and being less computationally expensive (Dosovitskiy et al., 2021).

#### Object detection and semantic segmentation architectures

Although more complex than YOLO, and arguably DETR, Faster R‐CNN delivers excellent results and requires only modest resources for training. For most object‐detection use cases in plant pathology, Faster R‐CNN will be the optimal choice. Mask R‐CNN extends Faster R‐CNN by adding the ability to predict a mask in a bounding box, enhancing its utility for semantic segmentation tasks. YOLO is most suitable for real‐time object detection but offers lower precision than Faster R‐CNN. It is not suitable for use in plant pathology unless inference time is of primary concern. DETR and Deformable DETR present a novel approach to object detection and offer competitive results (Zhu et al., 2021). However, implementing these architectures can be difficult and they require substantial GPU VRAM for training.

The choice of CV model architecture for a given project depends on a variety of factors, including data set size, signal‐to‐noise ratio, computational resources, mode of deployment, and accuracy requirements. However, at present, for most use cases in plant pathology, ResNet18, ConvNeXT Tiny, or Faster R‐CNN will yield the best results while minimising computational cost, risk of overfitting, and the financial cost of training.

### Image, batch, and layer normalisation

In a comparison of EVAL‐COVID (Gong et al., 2021) with other strong competitors like EVOCNN to detect COVID‐19 with evolved CNNs, it was shown that the overuse of batch normalisation (BN) can be deleterious to the training of DNNs for disease diagnosis. While BN often improves the training time of CNNs and can negate the need for small learning rates and dropout (Ioffe and Szegedy, 2015), its negative effect on the diagnosis of disease was also observed in Case Study 3 (Box 3).

Box 3 **Case Study 3: Disease detection and normalisation.**

Here, we conducted an ablation study with ResNet18 and ConvNeXt Tiny (Table 3) to assess the effects of image normalisation (IN), batch normalisation (BN), and layer normalisation (LN) in disease detection. Using BN in ResNet18 increased the training speed by 2.39 times, while IN slowed training by 1.74 times. IN did not affect training time in ConvNeXt Tiny. We also found that BN improved stability in training, as assessed by plots of training and validation loss. However, IN decreased the F1 score by 0.76% and 0.34% in ConvNeXt and ResNet18, respectively, and increased overfitting. The removal of BN in ResNet18 decreased the F1 by 1.92%, but ConvNeXt (in which BN is replaced with LN) had an F1 score 2.84% higher than ResNet18 with BN. Therefore, simply deactivating the BN layers in ResNet18 led to worse results in every metric. However, the use of LN instead of BN in ConvNeXt appears to have had no deleterious effect.

**Figure 2:**
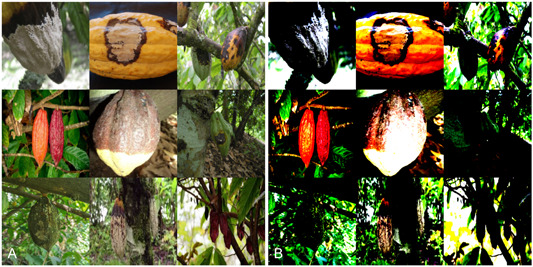
Image normalisation in the analysis of cocoa disease using computer vision and machine learning. (A) Original and (B) normalised images of cocoa pods showing various stages of frosty pod rot, black pod rot, and witches’ broom disease development. Note the effect of normalisation on one's ability to see disease symptoms. The normalisation of pixel values was carried out with the following means and variance values: mean: (0.485, 0.456, 0.406), variance: (0.229, 0.224, 0.225).

The removal of the IN transformation, which occurs prior to data input, improved the performance of both model architectures for the purpose of disease detection in all metrics, including training time and overfitting. These results are unsurprising if we consider the effect of image normalisation shown in Figure 2. Here, IN distorts the colour of the cocoa pods and obscures much of large lesions that are clearly visible in the original images. This effect may not prevent a CNN from identifying these objects as cocoa pods or trees by their shape, but it does obscure many subtle disease symptoms that are necessary for the detection of early disease development. The above ablation study was conducted with a data set of late‐stage disease, from which the Figure 2 images were sampled. So, if early disease detection were required, the differences between these methods may have been more pronounced. Additionally, BN has been observed to introduce unacceptable levels of error when the batch size is small (Wu and He, 2018). This is an important issue to consider for generative models, CV models with video or high‐resolution images, or when computational resources are limited.

**Table 3:** Results of an ablation study assessing the effects of image normalisations and batch normalisation on a model's ability to detect plant disease.

Image norm.	Batch norm.	Layer norm.	Train time (min)	Loss (%)	Accuracy (%)	Recall (%)	Precision (%)	F1 (%)
**ConvNeXt Tiny**								
No	—	Yes	1344	0.290	88.25	88.25	88.82	88.14
Yes	—	Yes	1368	0.322	84.51	87.51	88.14	87.38
**ResNet18**								
No	Yes	—	739	0.361	85.41	85.41	86.17	85.30
Yes	Yes	—	1088	0.380	85.14	85.18	85.68	84.96
No	No	—	1764	0.412	83.49	83.49	84.05	83.38

**Table 4:** Specifications and use cases for the hyperspectral cameras used in the following studies: Okamoto et al. (2007), Feng et al. (2018), Gutiérrez et al. (2019), Pan et al. (2019), Li et al. (2020), and Nguyen et al. (2020).

Make/model^a^	Task	Spectral range (nm)	Spectral bands	Spectral resolution (nm)
Resonon Pika II Vis‐NIR	Mango tree yield estimation	390–890	244	2
Headwall Nano‐Hyperspec w/pushbroom	Potato yield estimation	400–1000	272	6
Specim ImSpector N17E	Maize kernel vigour assessment	874–1734	NA	5
Specim ImSpector V10	Weed identification	400–1000	240	10
BaySpec OCI‐UAV‐1000 w/pushbroom	Nutrient assessment in rice	460–983	116	5
Specim ImSpector V10E	Disease monitoring in pears	328–1115	1002	2.8

^a^ Manufacturer locations are: Resonon (Bozeman, Montana, USA), Headwall Photonics (Bolton, Massachusetts, USA), Specim (Oulu, Finland), BaySpec (San Jose, California, USA).

Several state‐of‐the‐art generative models now omit BN entirely, while others replace it with weight normalisation or focus on fine‐tuning the momentum hyperparameter of BN layers (Vahdat and Kautz, 2021). As with simply removing the BN layers of a ResNet, reported above, replacing BN in ResNet with the alternative layer normalisation (LN) also results in worse performance (Wu and He, 2018). However, when the developers of ConvNeXt used LN as opposed to BN in their architecture, they observed that the model had no difficulty in training with this substitution (Liu et al., 2022). The BN momentum hyperparameter is a fixed weight applied to the running mean and variance calculations that are tracked during training and used during the application of BN at inference time. Thus, adjusting the BN momentum will not affect the effect training (Vahdat and Kautz, 2021). However, BN can cause the output of a layer to be slightly shifted during evaluation. A proposed solution to this is to optimise the momentum hyperparameter for each data set (Vahdat and Kautz, 2021).

In this section, we have listed a host of reasons why the unnecessary normalisation of data is to be avoided. While BN will shorten the training time for a CNN, it changes the input data in unpredictable ways, thus worsening classification results. However, at present, the best off‐the‐shelf CNN that is small enough to run on an older‐model smartphone is ResNet18. So, until a more suitable architecture becomes available, BN is unavoidable. We also show here and in Case Study 4 (Box 4) that optimisation of the BN momentum hyperparameter in ResNet18 leads to a slight improvement in the results of our cocoa disease detection model, that image normalisation should not be included in the training pipeline of a model that aims to make predictions from subtle colour features, and that excessive image input size is deleterious to classification accuracy.

Box 4 **Case Study 4: Optimisation of BN momentum and image size for cocoa disease detection.**

**Figure 3:**
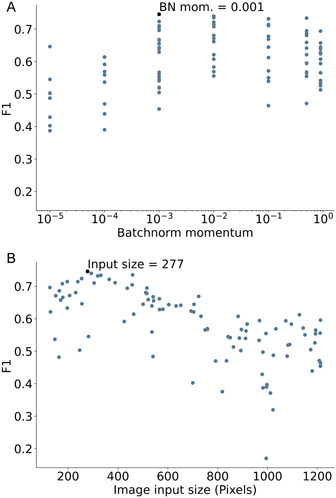
Results of a hyperparameter optimisation sweep training 100 ResNet18 models for disease detection in cocoa trees. This was performed with variable batch normalisation (Batchnorm) momentum (A) and a varying square image input size (B). The optimisation sweep randomly sampled from distributions of the two variables concurrently. Beginning with the ImageNet1KV2 weights, the models were trained on a data set of 1065 images of the following four classes: black pod rot (271 images), frosty pod rot (266), witches’ broom disease (92), and healthy cocoa (436). The optimised validation F1 score was 0.75.

While training a cocoa disease detection model, we ran a hyperparameter optimisation sweep using the Weights and Biases platform (WANDB; San Francisco, California, USA), which included the BN momentum hyperparameter and image input size (Figure 3). The model architecture used was ResNet18 (He et al., 2016), and the data set included the following four classes: black pod rot, frosty pod rot, healthy cocoa, and witches’ broom disease with a 90:10 training:validation set split and a training set size of 271, 266, 436, and 92 images, respectively. One hundred models were trained with these hyperparameters randomly sampled from predefined ranges (image size: 124:1224 pixels, BN mom.: 0, 10e‐5:0.9). We also used WANDB to run a random forest regression with the validation F1 as the dependent variable and the two hyperparameters as independent variables. From this, an importance score was calculated for each hyperparameter on a scale of 0–1. The highest‐performing model scored validation F1 = 0.75 and AUC = 0.87. Additionally, the per‐class F1 score for healthy cocoa was 0.88, showing a strong ability to detect non‐specific disease. While the importance of image size (0.694) is not surprising, the BN momentum score (0.306) is quite low. This casts doubt on the assertion above that optimisation of BN momentum can have much impact in lessening the deleterious effects of BN. However, this result and that of the optimised BN momentum value (0.001) (Figure 3A) suggests that this hyperparameter should be optimised, rather than relying on the default value of 0.1. Training the same model with a BN momentum set at 0.1 yielded an F1 score of 0.737, i.e., a 1.3% decrease relative to the optimised value.

This study also provides an optimised image input size for mid‐ to late‐stage disease detection, using ResNet18, of 277 × 227 pixels (Figure 3B), although this should be optimised for each use case. Image compression was previously said to have minor effects on disease detection (Barbedo et al., 2016), while elsewhere it is suggested that image compression should be avoided completely for small symptoms (Barbedo, 2016) or kept above an arbitrary 1 megapixel (1000 × 1000 pixels) (Steddom et al., 2005). However, with the present data set, which contains images of diseases at varying degrees of progression, using an image size greater than 277 × 277 was deleterious to the validation F1 score. This is in addition to the reduced image size providing faster runtime in training and inference, and a reduction in overfitting.

## DATA ACQUISITION AND MODEL TESTING

In this section, we review various interdisciplinary methods available for gathering a training data set and developing a suitable model. While the previous section concerned the theory of ML in CV, this section will focus on practicalities with respect to low‐cost solutions.

### Obtaining the required training data set

Training an image classifier to a high accuracy in a controlled laboratory environment is often a trivial task. However, such a model may perform poorly when presented with the challenges of the real world (Singh et al., 2020). For example, after training a leaf‐disease classifier on images taken in the field, the model performed with around 68% accuracy when tested against images taken in the lab (Ferentinos, 2018). However, when trained in the lab and tested in the field, the same model architecture performed with about 33% accuracy. This effect is likely due to the plain white background of the lab images causing the model to generalise poorly to real‐world applications. This exemplifies the importance of curating a realistic, high‐quality training data set. By naively training and releasing models that are trained on publicly available data sets, we risk exacerbating the problems of disease misclassification. At low frequencies, the effect of mislabelled, misleading, or uninformative data will have limited effect on the performance of a neural network. This feature of neural networks is largely an artifact of batch gradient descent and the learning rate (Motamedi et al., 2021), which act to greatly buffer the effect of infrequent misclassifications in the training data. At higher frequencies, these sources of error can have more serious consequences. The most obvious solution to this problem is to carefully curate, label, and annotate the training data. However, errors resulting from misclassification can be challenging to eradicate. For example, frosty pod rot, black pod rot, and witches’ broom disease in cocoa can all present with black or brown lesions on the pod, and both frosty pod root and black pod root can both coat a pod in white mycelium. This means that without sufficient training in plant pathology or access to diagnostic tests, one could easily mislabel these diseases. This problem can be solved by two means, which should be used in tandem: (1) by paying careful attention to detail and applying detailed knowledge of the pathogen in question, and (2) using tools and techniques from molecular biology and spectroscopy to better inform model development and subsequent disease detection. Such techniques/tools include DNA sequencing, real‐time quantitative PCR (qPCR), loop‐mediated isothermal amplification (LAMP), MultispeQ (PhotosynQ, East Lansing, Michigan, USA), and hyperspectral imaging (HSI).

#### Tools from molecular biology

DNA sequencing is now commonly used for the identification of cryptic species (Bickford et al., 2007; Ovaskainen et al., 2010) and plant pathogens (O'Donnell et al., 2015) and is an invaluable tool. Once sequenced, reads can be used to search previously categorised sequences with the Basic Local Alignment Search Tool (BLAST) from the National Center for Biotechnology Information (Boratyn et al., 2013) to identify a sample at the level of species or another taxonomic group. However, if we know which pathogen(s) we expect to detect, sequencing the whole genome is excessive. Rather, we can use loci such as the internal transcribed spacer (ITS) region of the nuclear ribosomal RNA genes, which are both highly conserved across taxa and highly variable between species. Such regions of the genome can be amplified using PCR or LAMP to detect a pathogen or identify it with relatively low cost and high accuracy. The ITS region is often sequenced on its own for near–species level identification or in concert with other loci for better specificity (Horton and Bruns, 2001). Such work with ITS is now ubiquitous in the molecular study of fungal ecology and phylogeny, while previous techniques relied on the morphology of fruiting bodies for identification (Horton and Bruns, 2001).

qPCR is used to detect asymptomatic disease across the agricultural industry (Luchi et al., 2020). Traditionally, PCR was unsuitable for portable operations or use in the field (Ray et al., 2017). However, rapid real‐time PCR in the field is now possible (Schaad and Frederick, 2002). Real‐time PCR can also be used to quantify the relative levels of a pathogen in plants (Horevaj et al., 2011). Information from such analyses could be extremely informative when fine‐tuning and assessing the performance of the models discussed here.

LAMP can be used in place of qPCR and has four key benefits: (1) it is considerably cheaper (£211or $256 USD for 100 samples) because a thermal cycler is not required; (2) it is fast; (3) the reagents do not need to be refrigerated; and (4) like real‐time PCR, there is potential for it to be used in the field. Like qPCR, LAMP can be used to quantify the relative amount of DNA present, as well as simply for detection. If detection is the only goal, colour‐ or turbidity‐based methods can be used to detect DNA presence by visual inspection. A drawback of this method is that any pre‐existing PCR primers cannot be used. This is because, while PCR primers are designed to amplify a specific region of complementary DNA, LAMP primers bind to multiple regions of the target DNA in a way that allows for the simultaneous amplification of multiple regions of the DNA.

While universal PCR primers for the ITS region exist, it may be necessary to design LAMP primers or species‐specific PCR primers for ITS or other regions. For a detailed discussion of the use of ITS amplification in fungal ecology and the potential pitfalls of specific ITS primer design, see Horton and Bruns (2001).

If novel primers are to be designed, the region of interest must first be sequenced, and if we aim to identify a currently unknown pathogen with BLAST, all of the DNA in a sample must be sequenced. Sequencing with the Oxford Nanopore Technology (Oxford, United Kingdom) MinION platform can be an ideal tool for this purpose, offering multiple features: (1) The Oxford Nanopore Technology field sequencing and library preparation kit allows for sequencing in the field, immediately after tissue samples are gathered, which eliminates the need for cold‐chain storage to avoid sample degradation. (2) It allows for high‐quality sequencing in countries where Illumina (San Diego, California, USA) sequencing is not available. (3) It is slightly cheaper than using the Illumina platform. (4) The long read length eliminates amplification bias (Goodwin et al., 2015). The avoidance of amplification bias is important for gene expression quantification, which is relevant to the discussion in the section “Inspecting informative features.” On the other hand, the MinION 1B requires a high‐spec computer and, at a cost of £98 or $119 USD per sample excluding library preparation, the use of this platform also remains too expensive for many projects.

#### Spectroscopy and HSI

Although not capable of specific disease diagnosis, the MultispeQ is an important low‐cost tool to consider in the context of disease detection in the absence of visible symptoms. This handheld plant phenotyping device can be used to indicate the non‐specific presence of plant disease at an extremely low cost (Kuhlgert et al., 2016). The MultispeQ operates similarly to photospectroscopy and measures environmental conditions such as light intensity, temperature, and humidity. It can also be used to measure photosystem II quantum yield, which is an indicator of plant health, and to detect non‐photochemical exciton quenching, which has been shown to have a significant negative correlation with disease index (Kuhlgert et al., 2016).

A highly informative technique that we can utilise in the prediction of plant disease with CV is to sample more continuously from the electromagnetic spectrum with HSI. As with the MultispeQ, HSI enables the detection of changes in the chemical composition of biological tissue in terms of conditions such as ripeness or disease status change (Bock et al., 2010). The term “spectral signature” is used to describe the pattern of electromagnetic radiation reflected by a subject. However, particularly in the case of biology, the term “signature” is misleading as biological samples often have highly heterogeneous reflectance spectra (Bock et al., 2010). All of the above‐mentioned CV studies applied ML techniques to RGB images. RGB images capture three discrete bands of the visible spectrum from 400–700 nm. Black‐and‐white digital images have two spatial dimensions and a single dimension that describes the darkness of each pixel on a scale of 0–255, whereas RGB images have three colour dimensions represented by values between 0–255, each describing the intensity of red, green, or blue light. Hyperspectral images, however, store a more complete reflectance spectrum for each pixel, while also maintaining spatial relationships. The spectral range of these images can be as wide as 300–2500 nm (Goetz et al., 1985).

Although the applications of hyperspectral photography have long been explored by the National Aeronautics and Space Administration, this technology is only now becoming affordable for use in industries such as agriculture. Commercially available cameras capable of capturing data from the 300–2500 nm range remain expensive, and more typically the cameras used only sample 330–1100 nm (Table 4). Despite the reduced spectral range of the cheaper cameras, they still provide orders of magnitude more data than RGB cameras, although much of these data are highly correlated.

The uptake of HSI has recently exploded in a host of fields including archaeology, art conservation, food safety, medicine, and crime scene investigation (Lu and Fei, 2014). Typical applications of HSI in agriculture include the estimation of yield (Gutiérrez et al., 2019; Li et al., 2020), assessment of vigour (Feng et al., 2018), remote weed identification (Okamoto et al., 2007), nutrient status (Nguyen et al., 2020), and disease monitoring (Pan et al., 2019).

The analysis of HSI data presents problems that are familiar to ML engineers and nowadays are solved routinely. These problems include the large size of HSI hypercubes, high dimensionality, high intra‐class variability, and high correlation between spectral bands. Many approaches have been taken to analyse these data and, for a long time, SVMs were the most widely used (Yue et al., 2015). Today, DNNs are commonly used to analyse these data as they are particularly well suited to the task of classification with HSI data. DNNs are able to isolate hidden and complex data structures, can utilise a great variety of data types, are flexible in their architectures and the complexity of the mathematical functions they can apply, and are ideally suited to distributed computing (Paoletti et al., 2019). As such, with the addition of dimension‐reduction techniques such as principal component analyses (Yue et al., 2015), the analysis of HSI data with DNNs, although more computationally demanding, becomes little more complex than such analyses of RGB image data.

While the field of CV is advancing at a rapid pace, so too are the fields of molecular biology and spectroscopy. The use of tools and knowledge from these fields will allow projects of various budgets to go beyond the simple application of CNNs to RGB images and, in doing so, model disease in greater detail with tangible biological explications of model behaviour.

### Model testing

#### The black box of DNNs

It is well known how poorly current CV models deal with unexpected edge cases and shifts in test data distribution (Schölkopf et al., 2021). However, in applying CV to plant pathology and agriculture, we encounter more cases than most ML practitioners where the test data does not align well with the training data. These problems arise routinely in CV from the effects of camera blur, image quality, or shifting camera angle. However, in plant pathology we must also contend with the perturbations of weather, climate, plant growth stage, crop variety, a plant's developmental response to growing conditions, and so on. While it remains contentious how robust of a fix techniques such as data augmentations or inductive biases may be to solve the former list of issues (Schölkopf et al., 2021), the latter issues will only be solved by truly understanding how our models are making predictions.

Although DNNs are still considered black box optimisers, much work has been done to understand their various facets and potential foibles. For example, each dense layer of a CNN has been shown to have distinct role in feature‐level extraction and generalisability (Yosinski et al., 2014), and the output of convolution layers have been visualised to show which physical features in an image were more exaggerated (Zeiler and Fergus, 2014). In a similar study, a host of predefined layer‐wise and neuron‐wise visualisation techniques were applied to a CNN that had been trained on images of plant disease (Toda and Okura, 2019). This work showed that the CNN in question was indeed using visible symptoms of disease that were similar to those used by human experts. Others have sought to learn how best to actively deceive or manipulate a DNN into misclassification. Working within the remit of cybersecurity, it was shown that image classifiers based on SVMs and DNNs could easily be deceived with a simple evasion algorithm (Biggio et al., 2013). This shows how brittle these classifiers can be and highlights the importance of adopting techniques that rely more heavily on causal inference, such as semi‐supervised learning (Peters et al., 2017) or semantic segmentation. It also highlights the importance of rigorous and conciliatory interrogation of models prior to deployment. At present, our methods of model evaluation are widely considered insufficient, and much more work is needed in this area.

#### Inspecting informative features

A key benefit of the use of CNNs is feature learning. This is the process by which a model will define for itself which features of a data set it considers informative (Voulodimos et al., 2018). In other CV algorithms, an engineer must handcraft descriptive features of a subject manually, using their expertise and/or diagnostic tools to guide them. In this latter case, pre‐processed data are used rather than raw data, as in a CNN. In the convolution layers of a CNN however, kernels and attention weights are applied to raw or augmented image data that emphasise informative physical features, and apply inductive biases and self‐attention before these data are passed to the dense layer(s) of the network (O'Mahony et al., 2020). We might assume that these physical features would include those that humans consider to be the obvious visible markers for plant disease, such as the presence of lesions on a leaf. However, it is likely that these networks will also identify markers that humans do not notice or cannot perceive, and may ignore some features that plant pathologists have long considered important. This provides us with the opportunity to learn more about how to identify disease early with human vision, CV, and molecular biology. Time‐series qPCR, transcriptome, or metabolome data can be used to identify the biological markers used by CNNs at the earliest moments of detection. This would allow for the validation of the image features used by the model. Such a biological explanation of the model's informative features would tell us whether the model were making correct inferences for what we consider to be correct reasons, or whether it were correct for spurious reasons, which would suggest a poor ability to generalise stemming from naive inductive reasoning. Such work may also highlight new ways to identify disease with or without ML or new ways of combating disease spread through phytosanitation, agrochemistry, or plant breeding.

In recent years, the combination of CNNs and transcriptomics in medical research has seen a surge in popularity. Such studies involve spatial transcriptomics (Chelebian et al., 2021; Xu and McCord, 2021), the identification of non‐small‐cell lung cancer subtypes (Yu et al., 2020), and the elucidation of the various functions of drugs (Meyer et al., 2019). In plant science, CNNs have been applied alongside transcriptomics in the investigation of gene regulation in *Arabidopsis thaliana* (L.) Heynh. (MacLean, 2019). However, the investigation of the black box nature of CNNs by means of omics appears to be completely absent from the literature.

Attention maps produced by software like Grad‐CAM (Selvaraju et al., 2017; Wang et al., 2019) are another way to inspect informative features of image data. Grad‐CAM produces an explanation for the decision that a model makes about a given image by visually highlighting the informative features of that image. Grad‐CAM is described as “gradient‐based” as it uses the gradient data that is fed into the last convolution layer of a CNN. This allows us to make assessments before the spatial relationships in the data are lost in the fully connected layers (Selvaraju et al., 2017). Alternative “reference‐based” systems, such as DeepLIFT, rely on back‐propagation (Shrikumar et al., 2017) or forward propagation (explanation map) (Ghosal et al., 2018), using a reference image that does not contain the feature of interest. Applying these methods to misclassified images can highlight why a model is performing suboptimally (Toda and Okura, 2019), as results produced with these methods have been shown to be highly correlated with assessments of plant disease made by human experts (Ghosal et al., 2018).

## A ROADMAP TO COMMERCIAL IMPLEMENTATION

Once you have developed, trained, and evaluated your model, it is time to begin the process of implementation. However, it is best to have considered and planned this step well ahead of time. There are several decisions made during development that may depend on the intended mode of implementation. For example, if the model is to be run on an edge device or smartphone, computation cost must be kept to a minimum. Likewise, if the model is to be made available via a rented server, reducing computational cost will reduce financial cost. Prior to training, choosing to use architectures such as ResNet18 and MobileNetV3 (Howard et al., 2019) will help to keep computational cost down and, after training, methods such as pruning and quantisation may reduce this cost further. While Google Colab (Google, Mountain View, California, USA; https://colab.research.google.com/) offers free limited access to GPUs for model training, the rental cost of a 16‐GB Nvidia V100 GPU (Nvidia, Santa Clara, California, USA), which would be the minimum needed to train a transformer model or large CNN, is $2.48 USD per hour. As such, developing and training such large models for days, or even weeks, can soon become expensive.

ONNX Runtime (Microsoft Corporation, Redmond, Washington, USA; https://onnxruntime.ai/) offers a huge array of tools to help accelerate, quantise, and deploy trained DNNs. Such models can be incorporated into Android or iOS apps using the phone's built‐in camera, and they can be deployed via the web, on edge devices such as a Raspberry Pi, or in embedded systems for drone mapping or smart irrigation. However, the operator schemas supported by ONNX Runtime must be considered here. For example, ConvNeXT, which uses Gaussian error linear units (GELUs) and stochastic depth, may cause problems as these operators are not yet supported. TensorFlow (Abadi et al., 2015) also offers a pipeline for model deployment, and the PyTorch toolkit for techniques such as quantisation aware training and model compression is maturing but presented difficulties when we attempted to use it. By contrast, the ONNX Runtime pipeline is extremely easy to use and supports all popular model formats, including PyTorch, TensorFlow, and SciKit Learn (Pedregosa et al., 2011). While the latest methods of pruning are reported to achieve a 30% reduction in the size of ResNet18 with only a 2% loss in accuracy on ImageNet (Solodskikh et al., 2023), this remains an active area of research, producing inconstant results. There is no guarantee that pruning will lessen computational cost. Techniques such as training‐aware pruning show promise but require further research.

For the implementation of object detection or segmentation models, we recommend the Detectron2 library from Facebook (Wu et al., 2019). This library incorporates Faster R‐CNN, Mask R‐CNN, and some new transformer models such as ViTDet, and offers a host of tutorials on the whole process from training to implementation.

## CONCLUSIONS

We describe here all of the tools necessary to develop highly optimised and robust ML models that use minimal computational power and provide real benefit to sectors that have more modest budgets. The application of these tools will allow us to break from the common trend in the ML industry, where expensive hardware is employed to develop complex and computationally expensive models to the detriment of improving training data quality.

With the application of off‐the‐shelf architectures to stock data sets, such as the PlantVillage data set (Geetharamani and Pandian, 2019), we can easily achieve prediction accuracy scores in the high 90% range (Thapa et al., 2020). However, such models have little value because they will not generalise to complex real‐world environments due to the simplicity of the training data and a high likelihood of overfitting.

We offer the following recommendations for the development of efficient, inexpensive, and robust CV models for plant pathology.

**Garbage in, garbage out**: The thoughtless application of advanced models to poorly labelled, simplistic, contaminated, or inappropriately transformed data will yield models that have little value in the field, with slow inference times, poor accuracy, and an inability to generalise. To avoid this fate, we should: (A) where possible, consult with specialists and utilise the invaluable tools from biology, chemistry, and spectroscopy to label data; (B) use the minimum appropriate image input size to improve runtime speed and help avoid overfitting; and (C) avoid needless data transformations such as normalisation, which can alter data in unreliable ways.

**The potential in training procedures**: Techniques such as semantic segmentation and semi‐supervised learning have potential to lessen both bias and variance in a model's predictions by promoting deductive reasoning over inductive reasoning. Additionally, appropriately scaled CNNs and evolved neural networks offer the potential to produce models with optimised runtime speed and improved generalisation ability.

**Robust and conciliatory interrogation of models**: While simpler modelling methods, such as SVMs, still have a role to play in modern CV, most of the models we employ for this purpose are exceedingly complicated and are prone to failing in equally complicated ways. Failure of a disease detection model resulting in an outbreak of disease could have very serious consequences. It is therefore vital that we rigorously test the models we develop to ensure that they are not prone to misclassification born of overfitting and naive generalisations. While metrics such as accuracy, F1, area under the receiver operating curve (AUC), recall, and precision are valuable, DNNs are often capable of learning to optimise these summary statistics indirectly, rather than learning to produce reliable predictions. Tools such as confusion matrices, cross validation, and explanation maps go much further in understanding the behaviour of CV models. However, it is important that we invest in the development of new and tailored means of understanding these models, such as the application of omics, as discussed in the section “Inspecting informative features.”

If we apply our wealth of knowledge and proven techniques from botany and agronomy to the acquisition of training data, the development of data‐processing pipelines, and the interrogation of trained models, we can produce applications with game‐changing potential. We are now only 27 years away from a predicted global population of 9.7 billion people (United Nations, 2022). Thus, with the devastating effects of the climate crisis already very much apparent, it is vital that we act now to build robust international infrastructure targeted at securing food supplies and eliminating extreme poverty. The techniques discussed here may enable us, as a community of growers, botanists, and ML developers, to help reduce poverty, improve the relationship between growers and the natural environment, and increase stability in the agriculture industry from the foundation up.

## AUTHOR CONTRIBUTIONS

J.R.S. conceived of this review, read and summarized the literature, and wrote the first draft of the manuscript. K.J.D. and D.W.F. continually reviewed and edited the manuscript. All authors approved the final manuscript.

## Data Availability

The image data, annotations, and link to the accompanying GitHub repository for Case Study 1 can be found at: https://osf.io/79kx3/?view_only=4a2c1dccee1a4baeb85de5002c702f10. For Case Study 2, the data used to train the initial supervised model, the .csv search terms file for the below web scraper, and the final semi‐supervised model weights can be found at: https://osf.io/h5gj7/?view_only=dbf9f245e21a41e185f5b73e718b4cad. The “contaminated” data used to train the semi‐supervised model were generated using the code at: https://github.com/jrsykes/Google-Image-Scraper. The custom code used to train both the initial model and the final semi‐supervised model can be found at: https://github.com/jrsykes/CocoaReader/blob/main/PlantNotPlant. The custom code and accompanying Readme.md used to conduct Case Study 3 can be found in the following GitHub repository: https://github.com/jrsykes/CocoaReader. The data for this study were scraped from the internet using the code in the following GitHub repository: https://github.com/jrsykes/Google-Image-Scraper. The location of the accompanying “.csv search terms file” is described below. The custom code to run the sweep in Case Study 4 can be found in the following GitHub repository: https://github.com/jrsykes/CocoaReader/tree/main/CocoaNet. The main script is titled CocoNetsweep_min.sh and the wandb config file is titled CocoaNetSweepConfig_min.yml. The data used to generate these results and the full wandb report can be found at: https://osf.io/2fw6g/?view_only=adc66ba66f83465a9e7b111515a60bf2.
